# Mind the gaps: overlooking inaccessible regions confounds statistical testing in genome analysis

**DOI:** 10.1186/s12859-018-2438-1

**Published:** 2018-12-14

**Authors:** Diana Domanska, Chakravarthi Kanduri, Boris Simovski, Geir Kjetil Sandve

**Affiliations:** 1 Department of Informatics, University of Oslo, Oslo, Norway; 2 K. G. Jebsen Coeliac Disease Research Centre, Oslo, Norway

**Keywords:** Assembly gaps, Reference genome, Statistical genome analysis, Colocalization analysis, Co-occurrence analysis, Region set enrichment analysis, Genomic overlap analysis, BED format

## Abstract

**Background:**

The current versions of reference genome assemblies still contain gaps represented by stretches of Ns. Since high throughput sequencing reads cannot be mapped to those gap regions, the regions are depleted of experimental data. Moreover, several technology platforms assay a targeted portion of the genomic sequence, meaning that regions from the unassayed portion of the genomic sequence cannot be detected in those experiments. We here refer to all such regions as inaccessible regions, and hypothesize that ignoring these regions in the null model may increase false findings in statistical testing of colocalization of genomic features.

**Results:**

Our explorative analyses confirm that the genomic regions in public genomic tracks intersect very little with assembly gaps of human reference genomes (hg19 and hg38). The little intersection was observed only at the beginning and end portions of the gap regions. Further, we simulated a set of synthetic tracks by matching the properties of real genomic tracks in a way that nullified any true association between them. This allowed us to test our hypothesis that not avoiding inaccessible regions (as represented by assembly gaps) in the null model would result in spurious inflation of statistical significance. We contrasted the distributions of test statistics and *p*-values of Monte Carlo-based permutation tests that either avoided or did not avoid assembly gaps in the null model when testing colocalization between a pair of tracks. We observed that the statistical tests that did not account for assembly gaps in the null model resulted in a distribution of the test statistic that is shifted to the right and a distribution of *p*-values that is shifted to the left (indicating inflated significance). We observed a similar level of inflated significance in hg19 and hg38, despite assembly gaps covering a smaller proportion of the latter reference genome.

**Conclusion:**

We provide empirical evidence demonstrating that inaccessible regions, even when covering only a few percentages of the genome, can lead to a substantial amount of false findings if not accounted for in statistical colocalization analysis.

**Electronic supplementary material:**

The online version of this article (10.1186/s12859-018-2438-1) contains supplementary material, which is available to authorized users.

## Background

Genome biology research relies on reference genomes to a large extent to map the high-throughput sequencing reads against known functional annotations [1]. The reference genome thus serves as a central entity that interlinks various genomic features [2]. Therefore, our current understanding of the genomes is greatly influenced by the completeness of the reference genomes [3]. However, because of the challenges associated with cloning and mapping certain highly repetitive and complex regions, the physical maps of the reference genomes of many species currently contain long-stretches of gaps [4]. For instance, a considerable proportion of the human genomic sequence (between 5–10%) remains poorly characterized to date. In the latest human genome build, hg38, around 200 Mbp mainly from centromeres and acrocentric short arms, and around 30 Mbp of interstitial gaps mostly in euchromatic sequences are currently uncharacterized [5]. The genome assemblies of non-model organisms contain a higher gap proportion than humans and model organisms (e.g., 6% gap bases in the genome of giant panda) [6, 7].

High-throughput sequencing reads are not expected to map against the assembly gap regions of the reference genome because of the lack of reference sequences. In many experimental settings, the little reduction in mapping rate due to the presence of assembly gaps is not a major constraint (owing to the relatively smaller size of the gap regions compared to the whole genome). However, not accounting for the presence of assembly gaps in the null model can be problematic in the statistical hypothesis testing of genomic colocalization.

In genomic colocalization analysis, the overlap or colocalization of genomic features is quantitated and subsequently tested for its statistical significance. The statistical significance of colocalization is often assessed by using either analytical tests (e.g., Fisher’s exact test) or tests based on Monte Carlo simulations/permutations. At the heart of either of the approaches is a null model, which is used to generate the expected null distribution of colocalization measure (under the assumption that there is no relation between the genomic features being tested). The definitions of null models vary depending upon how much of the real data characteristics they preserve [8, 9]. Accordingly, the null models can be considered as being simple/basic or as being conservative. There is no single null model that can fit all the analysis scenarios. Even a simple null model can be adequate in some scenarios if the assumptions of that null model are valid in the context of biological relevance. Nevertheless, choosing a null model that is closer to the characteristics of the real data being studied has been suggested to be a preferred way to avoid false findings in colocalization analysis [8, 9].Therefore, irrespective of the choice of the statistical testing approach or null model definition, one should ideally account for the assembly gap regions in the null model as it is still an inherent property of the real data. However, to our knowledge, no previous study explored the potential severity in terms of magnitude of false findings of not handling assembly gaps in the null model. The absence of such empirical evidence could partly be the reason why none of the current generation of colocalization analysis tools [10–18] handle assembly gap regions by default in the statistical testing. Furthermore, many technology platforms assay only a restricted part of the genome (e.g., exome sequencing, custom microarrays and so on), meaning that the resulting genomic tracks will be selectively depleted in certain regions. We refer to such depleted regions in general as inaccessible regions for a genomic track. Here, we hypothesise that the presence of inaccessible regions can introduce a bias and lead to false findings if not handled in statistical testing of genomic colocalization. We devise a simulation study that allows us to quantitate the effect of ignoring inaccessible regions in the absence of other signals.

## Results

### Overlap of public genomic tracks with genome assembly gaps

We performed a series of explorative analyses to understand the nature and extent of the intersection of genomic tracks with genome assembly gaps. For this, we downloaded large collections of public genomic tracks (hg19 and hg38) categorized by diverse experimental assays, including tracks of histone modifications, DNase I hypersensitive sites, and transcription factor binding sites in the K562 cell line for hg19 and DNase I hypersensitive sites for hg38 in Table 1. We then intersected the genomic tracks with assembly gaps of hg19 and hg38. Overall, we observed a trend of a very modest overlap with the assembly gaps and the overlap was unsurprisingly localized to the beginning and end portions of the gaps, rather than crossing over the middle portion of the gaps in Table 2. Assembly gap regions that overlapped the most with the public genomic tracks are on the chr4:40296396-40297096, chr7:139379377-139404377, chr3:194041961-194047251 followed by chr17:21976511-21976531. These findings rightly confirm that the sequencing reads do not map to the assembly gaps on hg19. This is not surprising, because unlike hg38, which contains sequence models for a large portion of the gaps, hg19 rather contains long-stretches of Ns in most of the gap regions, thus excluding the possibility of any read mapping.

**Table 1:** Track collection and aim of analysis

Tracks collection	Number of tracks	Genome	Source of Genomic tracks	Purpose
Histone modifications	477	hg19	ENCODE	Investigating the nature and extent of overlap of public genomic tracks with genome assembly gaps
DNase I hypersensitive sites	838			
Transcription factor binding sites (K562)	568			
DNase I hypersensitive sites	95	hg38		
Randomly selected tracks from the above collections	100	hg19	ENCODE	Understand the impact of not avoiding assembly gaps on the conclusions of statistical colocalization analysis
All tracks selected from the above collection	95	hg38

**Table 2:** Descriptive statistics of the intersection of public genomic tracks (counted relatively to the gaps size) with genome assembly gaps

Tracks collection	Genome	Avg size of genomic intervals relatively to the track size (bp)	Avg overlap with gaps (bp)	Avg overlap with the start region of gaps (bp)	Avg overlap with the end region of gaps (bp)	Avg overlap with the middle portion of gaps (bp)	Overlap hotspot	Overlap hotspot value (bp)
Histone modifications	hg19	7.43e-06	0.0014	0.0029	0.0023	0.0011	chr4:40296396-40297096	0.0591
TFBS in K562		3.89e-04	0.0046	0.0059	0.0053	0.0046	chr7:139379377-139404377	0.0448
DNase I hypersensitive sites		5.52e-06	4.49e-06	3.28e-05	1.21e-05	0	chr3:194041961-194047251	0.0007
DNase I hypersensitive sites	hg38	4.68e-06	0.0024	0.0026	0.0023	0.0024	chr17:21976511-21976531	0.9732

We further checked whether the amount of overlap of genomic tracks with assembly gaps is size-dependent. We observed that the overlap of public genomic tracks with assembly gaps increased with an increase in the average segment length of the tracks (Additional file 1: Figure S1).

### The impact of not avoiding the assembly gaps on the findings of statistical testing in colocalization analysis

**Fig. 1:**
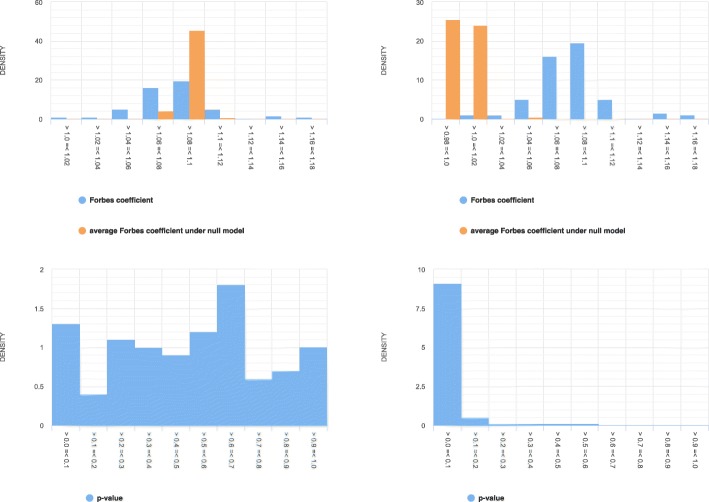
Distribution of the test statistic and *p*-values of colocalization analysis for a collection of 477 genomic tracks with 2113.82 bp average segment length for histone modifications (hg19). [**a** and **b** shows the distribution of *p*-values of the colocalization analysis with (left) and without (right) exclusion of assembly gap regions under the null model. **c** and **d** shows the observed test statistic and the average test statistic of the same tracks with (left) and without (right) exclusion of assembly gap regions under the null model. Note: Both values are higher than 1 because the computations were performed relative to the whole genome size.]

To understand whether avoiding/not avoiding the assembly gap regions in the null model would have an impact on the conclusions of statistical colocalization analysis, we contrasted the findings of MC-based permutation tests that either avoided or not avoided assembly gaps in the null model. For this, we first tested the significance of pairwise overlap of 100 pairs of genomic tracks (hg19) by not accounting for assembly gaps. Each of the pair of genomic tracks is comprised of a real track obtained from public repositories, while the other track was simulated to match the real track in its nature of not mapping to the assembly gap regions. The simulated tracks were generated with the same properties as real genomic tracks i.e. average segment length of intervals and number of elements and the same tendency to avoid assembly gaps. By simulating genomic tracks in this fashion, one from the outset can be sure that there is no dependence (association) between the real and simulated tracks except their shared avoidance of assembly gap regions (H0 is always true). However, when not accounting for assembly gaps in the statistical tests, the distribution of *p*-values of colocalization analysis is strongly shifted to the left, with H0 being falsely rejected after multiple testing correction (FDR <0.05) for 87 out of 100 tests (counted for tracks of histone modifications, Fig. 1b). This shows that ignoring gaps in the null model introduces a substantial risk of false discoveries. The bias of the analysis is also evident from a comparison of the distribution of observed test statistic values (number of bases overlapping) and the average values for the test statistic under the null model, where the average observed test statistic is higher than the average test statistic of the null model when ignoring assembly gaps (Fig. 1a, b and Additional file 2: Figure S2a, S2b, S2c, S2d).

**Fig. 2:**
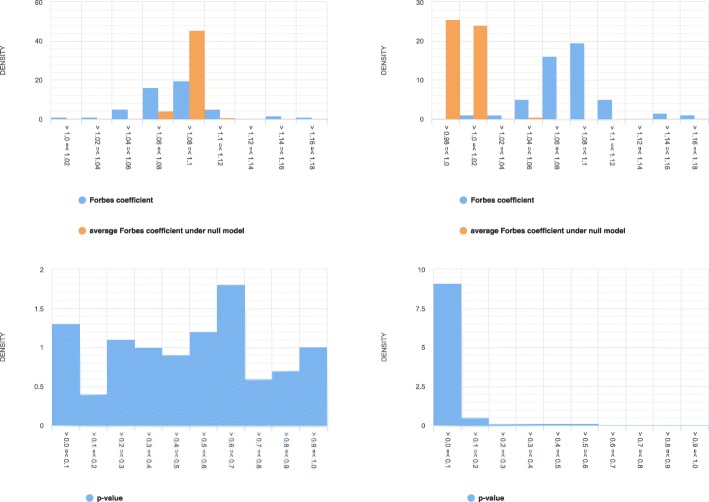
Relation between the *p*-values of colocalization analysis for a collection of N genomic tracks and the number of elements within each track of hg19 **a** for histone modifications (N=477) **b** for TFBS in K562 (N=568)

Furthermore, we repeated the statistical testing on the same dataset of 100 pairs of genomic tracks, this time by avoiding the assembly gaps in the MC-based null model. Accounting for the assembly gaps resulted in uniformly distributed *p*-values with no obvious shift in either direction (Figs. 1a, b and Additional file 2: Figure S2a, S2b and Additional file 3: Figure S3a, S3b). The comparison of the distributions of the observed test statistic and the average test statistic under the null model also substantiated that the null model is bias-free (Fig. 1c, d and Additional file 2: Figure S2c, S2c and Additional file 3: Figure S3b, S3c).

To examine further if a smaller proportion of the total gap size (relative to the genome size) would exclude the assembly gap bias, we repeated all the experiments discussed above on genomic tracks of hg38 (Additional file 4: Figure S5). We observed a similar trend of false findings as in the experiments with hg19, where the null hypothesis was falsely rejected for 83 out of 95 tests (∼87%) after multiple testing correction (Additional file 4: Figures S5b). This observation of nearly equal degree of false findings on hg38 tracks suggests that the assembly gap bias does not necessarily cancel out with a reduction in the total gap size. However notably, the total gap size of hg38 spans up to 5% of its total size whereas it was 7.6% in hg19. The degree of false findings might get reduced as the total gap sizes are minimized. Nevertheless, with the current versions of genome assemblies of different species, gap bias is still a technical confounder that is worth paying attention to avoid false findings in genome analysis.

## Discussion

Testing the significance of colocalization of genomic features is a common analysis approach in biomedical research. One common characteristic of genomic features assayed by current generation sequencing platforms is that the sequencing reads do not map to the gap regions of the reference genome, as also substantiated in this study. Similarly, some technology platforms assay only selective portions of the genome (e.g., excome sequencing, custom microarrays and so on). This selective depletion of reads in certain regions of the genome means that any subsequent statistical analysis should also carefully recapitulate this technical feature to avoid any potential bias. We hypothesized that failure to account for the selective depletion of certain genomic regions (here assembly gaps) is a technical bias that can confound the statistical testing in genome analysis. To test our hypothesis, we performed a pairwise colocalization analysis on a combination of real and synthetic datasets that have no true biological relation (association), using two different null models that differed only in whether or not they incorporate inaccessible regions. Since the datasets have no true association, statistical testing should not detect a statistically significant association, irrespective of its other assumptions about the data characteristics. However, our independent experiments on genomic tracks of both hg19 and hg38 showed that the null model definition which ignored this technical bias (assembly gap regions) detected a larger degree of false positives while the other null model that modeled the inaccessible regions resulted in a uniform distribution of *p*-values in an expected fashion. The bias was also visible when comparing the distributions of average test statistic under the null model (number of overlapping bases), where there is decreased overlap under the null model that did not model the technical bias. Such a trend of decreased overlap under the null model is not surprising since the null model is unaware of the technical bias and can distribute genomic elements in those regions also, whereas the real genomic tracks being tested are selectively avoiding those regions. Since the assembly gap regions span only a small portion of the genome (around 3–6% of the reference genome sizes typically), one cannot exclude the possibility of overlooking such a technical bias. However, the extent to which such a moderate bias may lead to a large number of falsely rejected null hypotheses (even after multiple testing correction) is noteworthy. The severity of false rejections will depend on the statistical power of the analysis - since the assembly-gap-ignorant null hypotheses are not technically true, they will all be rejected given enough data (even after multiple testing correction). Thus, the issue of assembly gap bias (or any other similar technical bias) may be highly problematic for datasets with many genomic regions, while it will be affecting conclusions to a lesser degree for datasets containing fewer genomic regions (see Fig. 2 and Additional file 5: Figures S4). The same point holds for other scenarios in which the tracks to be analyzed share a restriction to certain parts of the genome. This could, for instance, be datasets that are restricted to coordinates in transcribed regions or regions included on a custom chip or microarray. In such situations, data should in the null model be restricted to occur in the parts of the genome where observed data could technically occur. Note that although one should always aim for null models that realistically represents technical restrictions for the real data, the bias discussed in the present paper only applies when the datasets to be analyzed share a set of excluded regions (if data is unobtainable in certain regions only for one of the tracks, it will not lead to a systematic bias as described here). In this study, we have deliberately chosen to use a basic null model definition for both the null models (that differed only in whether they modeled the technical bias or not) because of the already complex study design. In this context, a “basic” null model definition is that, which do not closely preserve the real data characteristics such as genomic distribution (chromosomal locations), clumping tendencies and so on. However, we circumvented any potential effects of such a choice through our careful simulation design, where we know from the outset that the datasets that we are testing for association are not truly associated and do not have such complicating characteristics. Therefore, no null model definition should detect a true signal irrespective of its other assumptions about the real data characteristics. The findings of this study will not only benefit users of colocalization analysis methods, but are also crucial for the future endeavors in the direction of developing a robust bias-aware methodologies.

## Conclusions

This study demonstrates that failure to account for inaccessible regions, even when covering only a few percentages of the genome, can lead to a substantial amount of false findings when performing statistical testing of genomic colocalization. Since genomic colocalization analysis is often hypothesis-generating, users should be aware of such technical biases to avoid false findings.

## Methods

### Datasets

Throughout this study, we used large collections of genomic tracks that were either obtained from public repositories or simulated using a shuffling algorithm. Table 1 shows the genomic track collections used in this study. To investigate the nature and extent of overlap of genomic tracks with genome assembly gaps, we downloaded large collections of genomic tracks (hg19 and hg38) from ENCODE database. The downloaded genomic tracks are categorized by diverse experimental assays including tracks of histone modifications, DNase I hypersensitive sites, and transcription factor binding sites in K562 cell line for hg19 and DNase I hypersensitive sites for hg38.

**Fig. 3:**
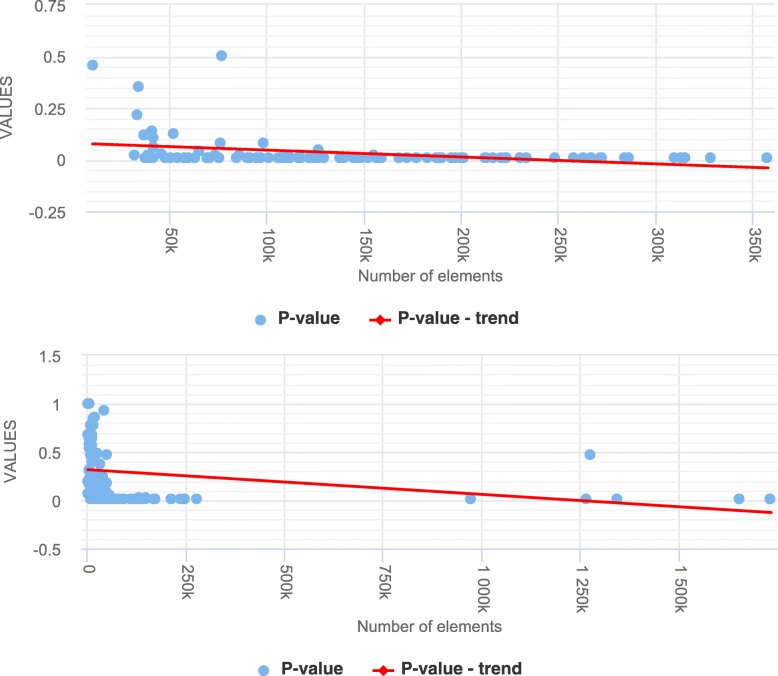
Schematic showing the study design. [To demonstrate the assembly gap bias if not accounted for in the statistical testing, we used two null model definitions that only differed in whether or not they avoided assembly gaps. For the pairwise colocalization analysis, we deliberately used a combination of real and synthetic track pairs to nullify any true biological association between them. The synthetic tracks were generated in such a way that they mimick the real tracks in terms of the genomic distributional properties as shown in the ellipse. The distributions of *p*-values, observed colocalization measures, average colocalization measures under the null models were examined to see if or not there is a bias.]

To understand the impact of either avoiding or not avoiding assembly gaps on the findings of statistical testing of colocalization analysis, we downloaded genomic tracks corresponding to histone modification sites (hg19 and hg38) from ENCODE database and randomly retained 100 tracks for the statistical analyses. For performing pairwise colocalization analysis (based on MC permutation tests) on the retained tracks of histone modifications, we simulated 100 synthetic tracks that match the histone modification tracks in terms of the number of genomic regions, the average length of the genomic regions, and the distribution of genomic regions across the chromosomal arms. The synthetic tracks are also deliberately simulated in such a way that they also avoid the genome assembly gaps, mimicking the typical nature of public genomic tracks. The synthetic tracks are simulated using a standard shuffling algorithm that distributes genomic elements uniformly across the genome by avoiding assembly gap regions, where chromosome and position are randomly chosen. For this analysis, we restricted the dataset size to 100 tracks because of the computational time of the MC permutation tests. The study design is shown on Fig. 3.

We further repeated the same experiments on 95 track pairs of hg38, where synthetic tracks were simulated to match the properties of 95 DNase I hypersensitive sites.

### Definition of null models

We have in our analyses considered two distinct null models that differ in whether genomic elements may occur in assembly gap regions, but are in other ways equal. Both null models preserve the number of genomic elements and their empirical size distribution, while assuming that these elements are distributed uniformly (without intra-track overlap) within the allowed parts of the genome (the full genome or the genome excluding assembly gaps for the two null model versions, respectively).

### Rationale behind using simulated genomic tracks to assess the gap bias

A pair of real genomic tracks from public databases can be associated in at least two different ways in the context of this study: (i) they both share the nature of avoiding the assembly gaps (as demonstrated in the first section of results section) and (ii) they both can share some true biological signal of genomic overlap (e.g., enrichment of DNase I hypersensitive exons near promoters). Hereafter, we refer to (i) and (ii) as “gap bias” and “true signal”, respectively. A naive null model can detect a significant association that has resulted because of either gap bias or true signal or both. This will be problematic if the detected significant association is not solely because of true signal (because even a weaker true signal could falsely appear stronger because of gap bias). The objective of this manuscript is to demonstrate that an over-optimistic finding (i.e. a *p*-value deemed statistically and biologically significant) could be observed even in the absence of any true signal between a pair of tracks, because of their shared nature of avoiding assembly gaps (gap bias). This can in principle be tested between pairs of real tracks. However, as pointed out above, real tracks can be associated in both ways: gap bias and true signal. Since the objective is to demonstrate the possibility of false positive findings even in the absence of true signal, one has to exclude the possibility of any true signal between the pairs of tracks being tested. Therefore, to exclude the true signal we generated synthetic tracks that essentially mimick real tracks except for their true signal.

### Tools

All plots and the information necessary for their reproduction can be found at https://hyperbrowser.uio.no/assemblygaps. All results can be reproduced using the redo-functionality provided by the underlying Galaxy system (https://hyperbrowser.uio.no/assemblygaps/u/hb-superuser/p/assembly-gaps).

## Additional files

Additional file 1 Overlap of public genomic tracks with genome assembly gaps of hg19 and hg38. Overlap of public genomic tracks with assembly gaps and average segment length of the tracks (a) for DNase I hypersensitive sites (hg19) (b) for histone modifications (hg19) (c) for TFBS in K562 (hg19) (d) for DNase I hypersensitive sites (hg38). Overlap of public genomic tracks with assembly gaps increased with an increase in the average segment length of the tracks. (PDF 60 kb)

Additional file 2 Distribution of the observed test statistic and the average test statistic under the null model for DNase I hypersensitive site (hg19). Distribution of the test statistic and *p*-values of colocalization analysis for a collection of 838 genomic tracks with 281.47 bp average segment length. (a) and (b) shows the distribution of *p*-values of the colocalization analysis with (left) and without (right) exclusion of assembly gap regions under the null model. (c) and (d) shows the observed test statistic and the average test statistic of the same tracks with (left) and without (right) exclusion of assembly gap regions under the null model. (PDF 70 kb)

Additional file 3 Distribution of the observed test statistic and the average test statistic under the null model for TFBS in K562 (hg19). Distribution of the test statistic and *p*-values of colocalization analysis for a collection of 568 genomic tracks with 3850.15 bp average segment length. (a) and (b) shows the distribution of *p*-values of the colocalization analysis with (left) and without (right) exclusion of assembly gap regions under the null model. (c) and (d) shows the observed test statistic and the average test statistic of the same tracks with (left) and without (right) exclusion of assembly gap regions under the null model. (PDF 69 kb)

Additional file 4 Distribution of the observed test statistic and the average test statistic under the null model for DNase I hypersensitive sites (hg38). Distribution of the test statistic and *p*-values of colocalization analysis for a collection of 95 genomic tracks with 150.35 bp average segment length. (a) and (b) shows the distribution of *p*-values of the colocalization analysis with (left) and without (right) exclusion of assembly gap regions under the null model. (c) and (d) shows the observed test statistic and the average test statistic of the same tracks with (left) and without (right) exclusion of assembly gap regions under the null model. (PDF 72 kb)

Additional file 5 Relation between the *p*-values of colocalization analysis for a collection of genomic tracks and the number of elements within each track. Relation between the *p*-values of colocalization analysis for a collection of genomic tracks and the number of elements within each track (a) for DNase I hypersensitive site (N=838, hg19) (b) DNase I hypersensitive sites (N=95, hg38). (PDF 39 kb)

## Abbreviations

MC: Monte Carlo
