# Supplementary material for: Mind the gaps: overlooking inaccessible regions confounds statistical testing in genome analysis
Source: BMC Bioinformatics. 2018 Dec 14;19:481. doi: 10.1186/s12859-018-2438-1 (PMC6293655; doi:10.1186/s12859-018-2438-1)
Supplement: Supplementary file 1 — Overlap of public genomic tracks with genome assembly gaps of hg19 and hg38. Overlap of public genomic tracks with assembly gaps and average segment length of the tracks (a) for DNase I hypersensitive sites (hg19) (b) for histone modifications (hg19) (c) for TFBS in K562 (hg19) (d) for DNase I hypersensitive sites (hg38). Overlap of public genomic tracks with assembly gaps increased with an increase in the average segment length of the tracks. (PDF 60 kb) [file 12859_2018_2438_MOESM1_ESM.pdf]

Additional Files

Additional file 1 — Overlap of public genomic tracks with genome assembly gaps of hg19 and hg38

Overlap of public genomic tracks with assembly gaps and average segment length of the tracks (a) for DNase I hypersensitive sites (hg19) (b) for histone modifications (hg19) (c) for TFBS in K562 (hg19) (d) for DNase I hypersensitive sites (hg38). Overlap of public genomic tracks with assembly gaps increased with an increase in the average segment length of the tracks.

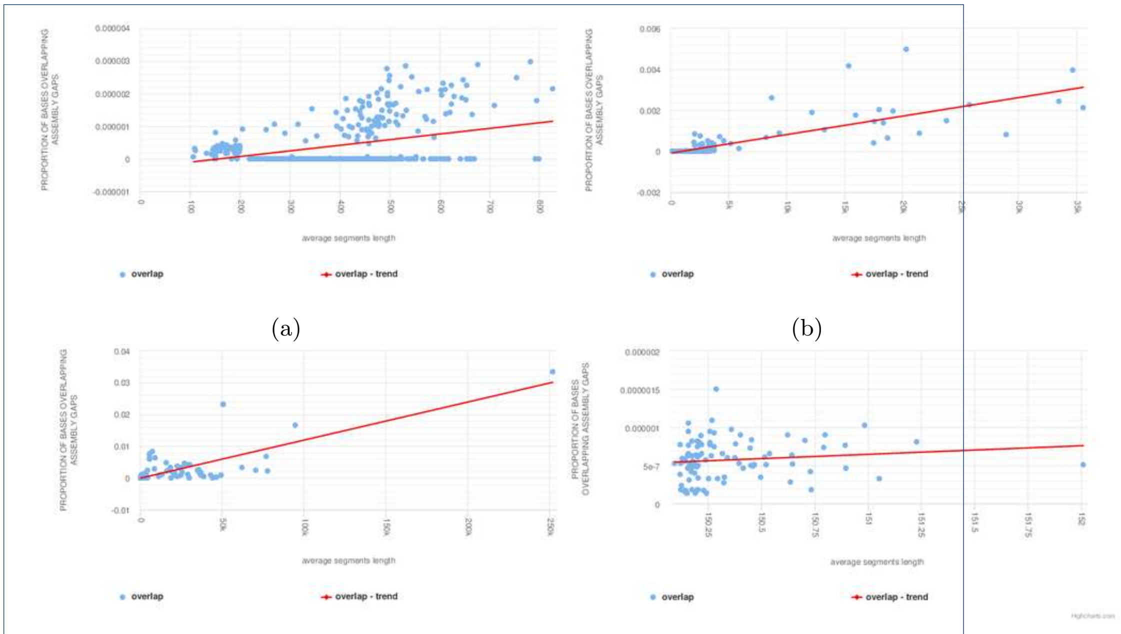

(a) (b)

(c) (d)

Figure S1
