# Supplementary material for: Mind the gaps: overlooking inaccessible regions confounds statistical testing in genome analysis
Source: BMC Bioinformatics. 2018 Dec 14;19:481. doi: 10.1186/s12859-018-2438-1 (PMC6293655; doi:10.1186/s12859-018-2438-1)
Supplement: Supplementary file 3 — Distribution of the observed test statistic and the average test statistic under the null model for TFBS in K562 (hg19). Distribution of the test statistic and p-values of colocalization analysis for a collection of 568 genomic tracks with 3850.15 bp average segment length. (a) and (b) shows the distribution of p-values of the colocalization analysis with (left) and without (right) exclusion of assembly gap regions under the null model. (c) and (d) shows the observed test statistic and the average test statistic of the same tracks with (left) and without (right) exclusion of assembly gap regions under the null model. (PDF 69 kb) [file 12859_2018_2438_MOESM3_ESM.pdf]

Additional file 3 — Distribution of the observed test statistic and the average test statistic under the null model for TFBS in K562 (hg19)

Distribution of the test statistic and p-values of colocalization analysis for a collection of 568 genomic tracks with 3850.15 bp average segment length. (a) and (b) shows the distribution of p-values of the colocalization analysis with (left) and without (right) exclusion of assembly gap regions under the null model. (c) and (d) shows the observed test statistic and the average test statistic of the same tracks with (left) and without (right) exclusion of assembly gap regions under the null model.

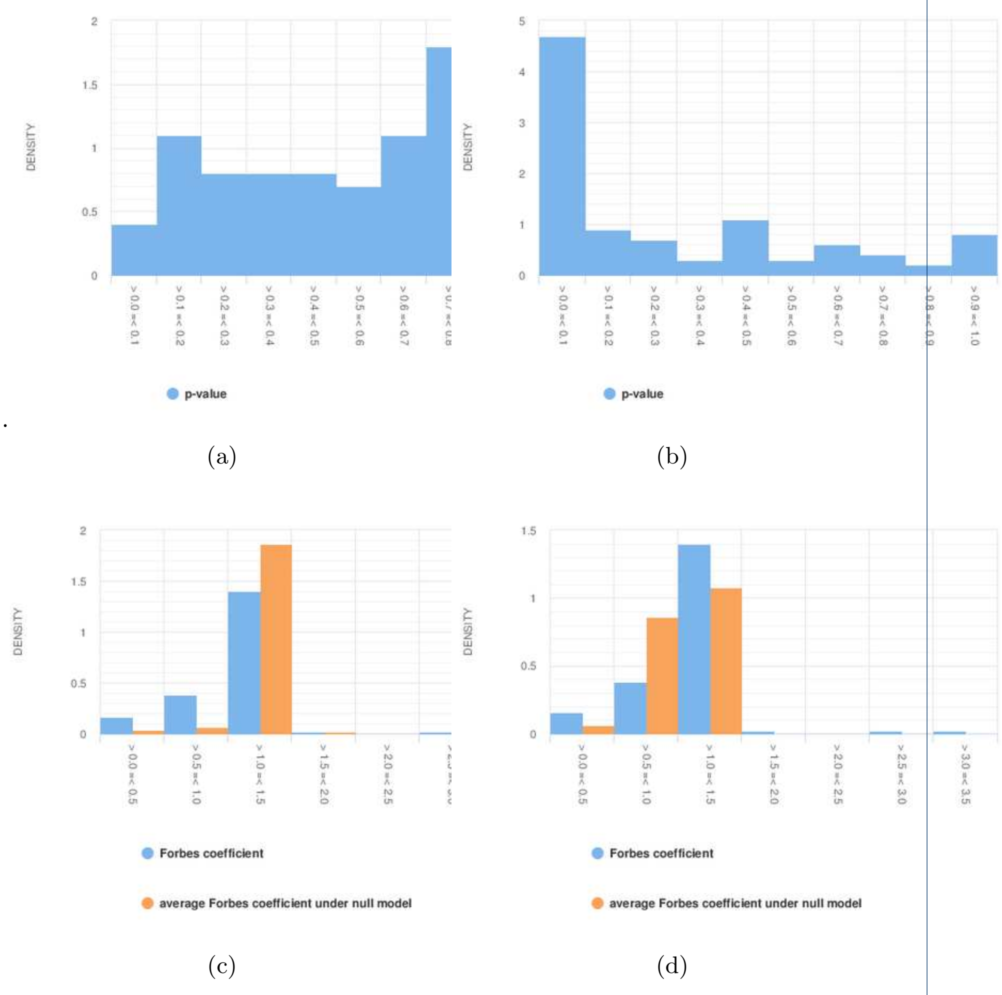

Figure S3
