# Supplementary material for: Mind the gaps: overlooking inaccessible regions confounds statistical testing in genome analysis
Source: BMC Bioinformatics. 2018 Dec 14;19:481. doi: 10.1186/s12859-018-2438-1 (PMC6293655; doi:10.1186/s12859-018-2438-1)
Supplement: Supplementary file 5 — Relation between the p-values of colocalization analysis for a collection of genomic tracks and the number of elements within each track. Relation between the p-values of colocalization analysis for a collection of genomic tracks and the number of elements within each track (a) for DNase I hypersensitive site (N=838, hg19) (b) DNase I hypersensitive sites (N=95, hg38). (PDF 39 kb) [file 12859_2018_2438_MOESM5_ESM.pdf]

Additional file 4 — Relation between the p-values of colocalization analysis for a collection of genomic tracks and the number of elements within each track

Relation between the p-values of colocalization analysis for a collection of genomic tracks and the number of elements within each track (a) for DNase I hypersensitive site (N=838, hg19) (b) DNase I hypersensitive sites (N=95, hg38).

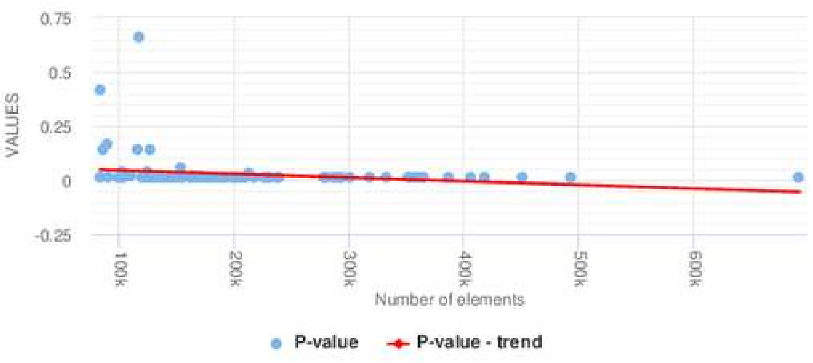

(a)

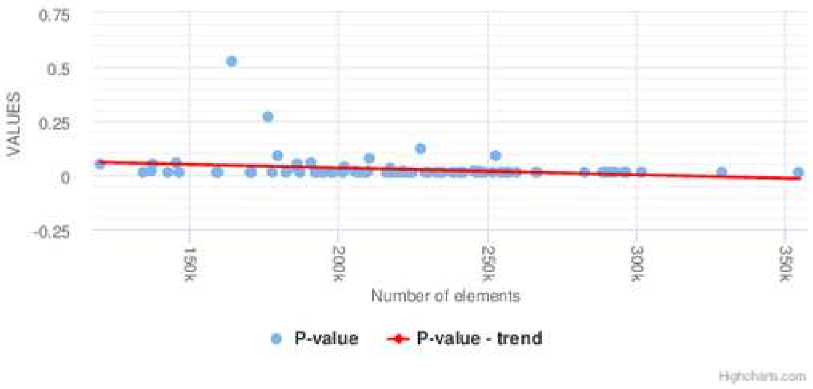

(b)

Figure S4
